# Nursing Section

**Published:** 1904-01-16

**Authors:** 


### The Hospital.

### Nursing Section.

Contributions for this Section of "The Hospital" should be addressed to the Editor, "The Hospital" Nursing Section, 28 & 29 Southampton Street, Strand, London, W.C.

No. 903.—Vol. XXXV. *SATURDAY, JANUARY* 16, 1904.

### Notes on News from the Nursing World.

### THE QUEEN AND THE NURSES OF BAKEWELL INFIRMARY.

During the stay of the King and Queen at Chatsworth, the Queen, accompanied by Princess Victoria and the Duke and Duchess of Devonshire, visited the workhouse at Bakewell. After inspecting the main building, the party were conducted to the infirmary, and met at the entrance by the nurses. They then took off their wraps and proceeded through the various wards. The Queen requested the nurses to give her particulars of each individual case as she arrived at the bed, how long each patient had been in the infirmary, the nature of the disease, and the diet. In the maternity ward her Majesty asked Mrs. Ponsford, the matron, if a certain patient had sufficient clothes for the baby to go out with when the mother desired to take her discharge, and upon being informed that in most instances the matron had to provide the mothers with the necessary articles, she gave Mrs. Ponsford half a sovereign for that particular woman, requesting her to see that the little one was sent out comfortably clad. The Queen conversed with the nurses and patients quite freely, and expressed herself well pleased with everything. On leaving she shook hands with each of the nurses.

### PRINCESS CHRISTIAN AT THE ROYAL FREE HOSPITAL.

On Wednesday afternoon Princess Christian visited the Royal Free Hospital, of which she is President, and distributed the gifts provided by her to 150 patients and 50 sisters and nurses. A bouquet of white tulips was presented to her Royal Highness by a little girl who has just recovered from a serious operation, and one of yellow daffodils was offered to Princess Victoria of Schleswig-Holstein by a little boy-patient. Her Royal Highness then proceeded to Calthorpe Ward, where patients, nurses, students, and many members of the medical staff were assembled. A large tree, lighted by electricity, had been placed on a raised platform, the presents being arranged on two tables. Both presents and decorations for the tree had been personally chosen by the Princess, who was assisted in the task of distributing them by her daughter, with Miss Wedgewood (the matron), Dr. Sainsbury, and Mr. A. Boyce Barrow. Many of the presents were of considerable value, and included some choice specimens of black Wedgwood, Crown Derby, and Florentine china, while among the gifts to the nurses were some water-colour drawings and several pieces of exquisite embroidery. Each sister received a photograph of the Princess, framed in green leather, and signed "Helena." After the distribution a vote of thanks to Her Royal Highness was proposed by Mr. Holroyd Chaplain. The Princess subsequently partook of tea and visited the wards.

### SIR WILLIAM BENNETT ON MODERN NURSING.

The address on Some Aspects of Modern Nursing, especially in private practice, by Sir William Bennett, of which we report the first portion to-day, will be read with general interest. Delivered to the nurses of St. George's Hospital last week, it contains much that merits the serious consideration of members of the profession everywhere. Sir William Bennett's wide knowledge and great experience invest his opinions with weight, and we feel sure that in this instance they will be taken to heart by many outside the institution in which they were uttered. In the early part of his address Sir William lays stress upon the short period of time a nurse, more especially a surgical nurse, can work pointing out that it behoves those who have no private income to fall back upon to qualify themselves for maternity work, in order that when they have become middle-aged nurses they may still be able to continue their profession in another branch. He also maintains that "a highly-educated nurse in the transcendental sense is not always an efficient nurse from the point of view of the surgeon, the physician, or the practitioner," and while freely admitting that the nurses of to-day are in some respects very superior to those of the recent past, contends that greater refinement, as well as higher education, may involve disadvantages as well as advantages, at any rate to the patients. Some very plain words on a burning question will be found in the concluding portion of the address which will be published next week.

### THE "CLAIMS" OF ARMY NURSING SISTERS.

It has been suggested, in the supposed interests of army nursing sisters, that their "claim" to be allotted on their retirement from the service residences similar to the suites of rooms in Hampton Court Palace, should be put before the King. We are quite sure that his Majesty is already fully aware of the value of the work done by army nurses, and does not therefore need to be provided with any information from the department. But whatever recognition he may, at any time, think proper to bestow, he is not at all likely to present them with a white elephant. The people who talk about army nursing sisters being assigned suites of rooms like those at Hampton Court can hardly be aware that, even if the suggestion were practicable, the keeping up of such residences would be utterly beyond their reach. Moreover, we object to the use of the word "claim" in the matter. Army nursing sisters receive their salaries and their pensions, and anything further would be favours conceded. We do not think that, in any event, it would be desirable for a number of retired nurses to live in a sort of little colony of their own. After they have finished their nursing experiences, they do not desire to remain permanently in a nursing atmosphere. The case of widows of officers, who often have families, is not analogous.

### DEATH OF A HONGKONG MATRON.

The Alice Memorial Hospital, Hongkong, has recently sustained a severe loss in the death of its matron, Mrs. Stevens, who succumbed to sprue on December 6, after a long and tedious illness. Mrs. Stevens had done much towards bringing the nursing up to its present standard. She had been unwearying in her endeavours to educate the Chinese nurses upon Western principles, and to inculcate in them the compassionate interest for their sick fellow country-men which is usually conspicuous by its absence in far Cathay. It speaks volumes for the success of her efforts that the work during several months was carried on by her pupils without trained European help, the direction of the hospital being undertaken by ladies with a slight knowledge of nursing under the supervision of the doctor in charge. Mrs. Stevens had a wide circle of friends by whom she will be greatly missed and mourned.

### CROYDON GUARDIANS AND THE MATRON'S SIGNATURE.

We are glad that, as reported in our columns last week, a late probationer at the Croydon Workhouse Infirmary has raised the question why her certificate of training does not bear the matron's signature. The omission of which she complains dates from the time of a difficulty which arose over the signing of some certificates when Miss Julian was matron. The Guardians, apparently, thought that the best way of preventing its recurrence was to issue a fresh form of certificate without any provision for the matron's signature. Miss Julian's retirement with compensation, and the appointment of a new matron, did not affect the position; and since the new form was printed no certificate given has borne the matron's name. The Croydon Guardians can hardly realize the harm they are doing the Infirmary under their charge as a training school by persistence in this policy. It must be obvious that the character and capacity can be more accurately gauged by the matron than by any other person, and so long as she does not sign the certificate, the probationers at Croydon Infirmary are placed at a serious disadvantage compared with those who are trained in schools where the usual course is pursued. It follows that young women who desire to obtain the certificates that will be most helpful to them in their nursing career will avoid an institution which, under existing circumstances, does not supply that want.

### A NEW YEAR'S CHARGE TO NURSES.

The Bishop of Kensington visited St. Mary's Hospital, Paddington, on Wednesday evening last week, when a special service for the nursing staff was held in the hospital chapel. The sermon was practically a New Year's charge to the nurses, and was founded upon the words "Thy will be done on earth as it is in heaven." Dr. Ridgeway impressed upon the nurses the fact that the prayer, though given for the use of others by Our Lord, was His own prayer also. He was always doing His Father's will, and the doing of God's will was the duty which was imposed upon nurses and those who ministered to the sick. The most commonplace and menial work—the work that seemed at times most monotonous—might be a means of glorifying God. The Bishop emphasised the importance of a little time of quiet communion with God as a help to the achievement of real success in the nursing profession.

### CHELSEA HOSPITAL FOR WOMEN.

On Saturday afternoon the patients of Chelsea Hospital for Women had their annual treat in one of the wards, which was decorated for the occasion. Palms and plants surrounded the small stage at one end of the room, and the patients, in gay dressing-gowns with pink ribbons in their hair, were grouped round in beds or easy chairs. The nursing staff and a few guests were also present. The first part of the programme consisted of the presentation, by Dr. Comyns-Berkeley, of gifts to the patients. Each member of the nursing staff having also received a parcel the entertainment began. There were some amusing songs and dialogues by two nigger minstrels, one of whom gave clever imitations of various familiar sounds. Then came an exhibition of living marionettes, in which Dick Turpin, a sea pirate, a Highlander, and other striking personalities danced and sang to the great delight of the onlookers. At the close Mr. Beaman said that thanks were due to Mrs. Morgan and the Ladies' Committee for arranging and organising the entertainment which had given so much pleasure to all who were present; also to Dr. and Mrs. Fenton for their kindness in providing the pretty and useful presents for patients and nurses, to Lady Edwardes Moss for providing the ladies' orchestra on December 7th, to Mrs. A. W. Foster for the Christmas fare of turkeys, and to Miss Room for the Sunday-school carol singers. The wards were prettily decorated with violets, fresias, and chrysanthemums, intermixed with smilax and asparagus fern, fairy lamps of many colours being freely used. These were also placed along the corridors where they had a very pleasing effect.

### BELFAST UNION INFIRMARY.

On Tuesday last week an "At Home" was given by the nursing staff of the Belfast Union Infirmary in the new home, with accommodation for 140 nurses, recently built by the Board of Guardians, who do their best to promote the comfort and welfare of their nurses. The concert-room and large dining-hall were beautifully decorated by the staff, who assisted the Lady Superintendent in receiving and entertaining the guests, and also arranged and carried out the musical part of the programme. The staff and guests numbered 280, including the resident and visiting medical staff. An enjoyable evening was spent, and the nurses are to be congratulated on the success of their first entertainment.

### A FANCY DRESS CONCERT BY NURSES.

The nurses of St. Luke's Hospital, Halifax, gave their second annual concert to the patients on New Year's Eve. An excellent programme was gone through, the various songs and choruses being taken from light operas, including "Gondoliers," "Country Girl," and "Florodora." The nurses donned fancy dress costumes of the Roman and Grecian styles, the variety and combination of colours having a charming effect. In addition to the musical part of the programme were "Tableaux Vivants," into which many of the nurses entered with enthusiasm. Dr. Sharp, Miss Ward, and Nurse Wilkin received many compliments on their successful arrangement of the tableaux, which displayed much taste, care, and skill, and the services of Nurse Edwards, as general manager of the musical part, were warmly acknowledged.

### ROYAL COUNTY HOSPITAL, RYDE.

THE nurses at the Royal County Hospital, Ryde, were especially busy this year in organising Christmas entertainments for the patients under their charge. On Boxing Day a concert was given by the probationers, and on the following Monday the nurses gave a display of tableaux and waxworks in the outpatients' department. The ward decorations were particularly original. In the women's ward was a pretty and effective grotto representing a frozen pond in a snowstorm. In the men's ward, in a recess, was a gipsy's tent, great amusement being caused when the gipsy arrived to tell fortunes, whilst in the children's, beside a gaily-lighted Christmas-tree, was a huge snowball, filled with presents for the little ones. On Christmas day the nurses were all invited to tea with the matron, and afterwards music, dancing, and games were much enjoyed.

### HERTFORD HOSPITAL, PARIS.

THE general experience of English nurses in the French capital is that Christmas is very little observed, but this year it was kept in true British style at the Hertford Hospital, Paris. On the day itself the hall and the wards were beautifully decorated, and after the patients had finished their dinner of roast beef, poultry, and dessert, tables were laid in the large hall for about twenty guests, the matron, house surgeon, and the nurses. Some of the guests were artistes from the Grand Opera, and from 3 to 5.30 they gave an entertainment of vocal and instrumental music for the patients, the nurses also much appreciating the treat. On Boxing Day, through the kindness of the Committee and many outside friends, a huge Christmas tree lighted by electricity and laden with presents for all was provided, toys being distributed also to the children. At the close of the evening one of the visitors expressed the feelings of all present, and thanked the matron, doctor, and nurses for all the pleasure they had received. On New Year's Eve the hospital staff acted a couple of short plays. When midnight struck the party adjourned to the front of the hospital and sang "Auld Lang Syne."

### AN OFFICIAL OF THE UNION.

WE are not surprised to learn, through the courtesy of Mr. Armstrong, the master of Thame Workhouse, that the person who was sentenced at Oxford a few days ago to six weeks' hard labour for brutal behaviour to an aged woman in the sick ward of that institution, though called Nurse Jones, does not hold a certificate. She was merely "an official of the union." Her shameful misconduct is, therefore, no reflection upon a noble profession. But the incident only emphasises the importance of employing a trained nurse to attend upon the sick poor both by night and by day. We cannot readily believe that any woman holding a hospital or infirmary certificate would beat a patient suffering from paralysis with a stick. After their experience of the application of the cheap and nasty principle, the Guardians of Thame Union will, perhaps, recognise the wisdom of putting a certificated nurse, instead of an official of the union, on duty in the sick ward.

### A NURSE'S SUCCESSFUL ACTION FOR LIBEL.

THE second trial in the Birmingham County Court of the action brought by Miss Mildred Davis, a nurse formerly attached to the Handsworth Nursing Home, ended last week in a manner which we think must be satisfactory to the plaintiff. It is true that Miss Davis claimed £300 damages for the statement of Mrs. Thompson that during her attendance on her the nurse had disgraced herself, and that the plaintiff has only been awarded £50. But all costs have also been given her, and, much more important than this, her character has been conclusively vindicated. In the cross-examination of the nurse it was sought to show that she broke the rules of the Home to which she was attached by asking for stout, that she obtained goods and had them placed to Mrs. Thompson's account, that she opened a letter sent by Mr. Thompson to his wife, and that she divulged professional secrets. As to the first allegation, Miss Davis admitted that during the time she attended Mrs. Thompson she had consumed four bottles of stout because she was not well, but she emphatically denied all the other charges, and said that she would have agreed with the term "disgraceful" if they had been true. The jury believed her, and we trust that she will now be able to resume her position at the Handsworth Nursing Home, which, in consequence of the allegations made about her by Mrs. Thompson, she had been compelled to resign.

### MIDWIVES INSTITUTE.

DR. MARY ROCKE will lecture on "Infant Feeding and Infants Diseases" at the Midwives Institute, 12 Buckingham Street, Strand, on Monday, January 25th, and Monday, February 2nd. The lecture will begin each afternoon at half-past three.

### SHORT ITEMS.

THE Queen has sent her annual subscription of £5 to Queen Charlotte's Hospital, and the Princess of Wales has also sent a donation of £5.—The children at the Hospital of St. John and St. Elizabeth, Grove End Road, N.W., had a Christmas Tree on Thursday last week, lighted with electric lights, which as well as the elegant illumination of the room, was the work of a generous benefactor. A piano has also lately been given to the ward.—The Zenana Bible and Medical Mission will hold a Thanksgiving Day on Tuesday, January 26th, in Morley Hall, George Street, Hanover Square, and the managers hope that before then the remaining £4,000 still needed to meet the cost of increased medical and Zenana work will be sent in.

### The Nursing Outlook.

" From magnanimity, all fear above;
From nobler recompense, above applause,
Which owes to man's short outlook all its charm."

### THE CARE OF THE INSANE.

THE meeting to discuss the first of the proposed nurse-registration bills removed one of many defects by giving representation to the Association of Asylum Workers—a body which consists of about 4,000 mental nurses of both sexes, 200 medical men engaged in asylum work, and 200 "associated members," such as stewards, retired matrons, and so on. Now in the present panic about the physical degeneration of the race, there is a tendency to neglect the equally serious mental deterioration which is assuming alarming proportions. Note the following figures from the census returns :—

*Numbers of Insane in England and Wales.*

| | | |
|---|---|---|
| In 1871 | there were | 69,019 |
| In 1881 | " | 84,503 |
| In 1891 | " | 97,383 |
| In 1901 | " | 132,654 |

And the greatest increase of all was reported for 1902 when nearly 500 people a week became insane ; the total of new cases for the year being 22,581.

But now let us look to the more hopeful side of the case. Though mental disease is thus terribly on the increase, its study and cure is proceeding proportionately. Whereas in the old days one saw metaphorically over every asylum " all hope abandon, ye who enter here"—now patients go almost as readily in search of mental as of physical help from medical science. And the acknowledgment that a man has had to seek temporary refuge in an asylum is no longer regarded as a positive ban to his future.

Now, exactly as in the hospital, the care of the nurse is as necessary for the patient as the skill of the doctor ; so in the asylum the chance of cure rests largely on the tact and patience of the attendant. We admit this used not to be so, that in old days the chief duty of the attendant was to restrain, but in this age of certificated mental nurses things are very different. Only last week the following significant advertisement from a county asylum appeared in our pages :—

PROBATIONER NURSES REQUIRED. Three years' training in general and mental nursing is provided for ladies desiring to specialise in this direction. As the nursing staff is composed of persons of superior social status it is useless for others to apply.

Salary commences at £20 per annum, with Board, Lodgings, Washing, and Uniform.

And in his lectures on "Mental Diseases," Dr. Jones has called attention to the present combining of hospital and asylum nursing, and given particulars of the knowledge necessary for those nurses who undertake the care of the insane. Also let it be remembered that the Medico-Psychological Association hold twice a year an examination for mental nurses who have served for three years in an asylum and who are recommended for examination by their own medical officer. At the last examination held there were 766 candidates, of whom 174 failed. The questions included the following :—

"4. What symptoms should you expect to find in a case of disease of the respiratory organs ? 5. Mention the more common forms of insanity, and state briefly the distinctive features of each. 6. What is a fracture ? What is the difference between a simple and compound fracture ? Which is the more serious injury ? Why ? 7. What are the chief kinds of sick diet and why are they given ?"

And, in conclusion, the Scotch Commissioners in Lunacy state with regard to Stirling District Asylum :—

"Another, and perhaps the most important, of the new administrative methods is the great extension of the employment of female nurses in the male wards. Out of a total staff of 43, 19, or nearly one-half, are women. Eighteen men and 14 women during the day, and 6 men and 5 women during the night, share the responsibility of the care of the male patients. It is admitted by those who have had the largest experience, both in this country and abroad, that female nursing is preferable for sick and infirm men, and it is also believed by many that the presence of women in asylum wards obviates, proportionately to their number and influence, those regrettable personal conflicts which tend to occur from time to time when insane men are wholly attended by individuals of their own sex. There are other favourable considerations, but the two mentioned are in themselves sufficient to justify a commendation of the system."

Surely enough has now been said to prove that the large number of skilled attendants on the insane are a recognised force in the nursing world and do honourable work for the community ? They are not so much before the public as the hospital nurse, they are not so spoilt by sentimental admiration. But that is no reason why they should not be applauded for their steady progress, and the capable way in which they are marching with the times and rising to the new developments of our complex civilisation. "In quietness and confidence shall be your strength" is more true for the mental attendant than for any other worker, but all the same it is ignorance or arrogance for hospital nurses to forget the claims of what promises to become the largest branch of their profession, both in numbers and in importance. In fact we seem to be drifting fast towards that state of affairs foretold by the Frenchman, when the inhabitants of England will inclusively consist of inmates of institutions and officers and committees for the working and governing of those same institutions!

### Sir William Bennett on Modern Nursing in Private Practice.

AN Address upon some Aspects of Modern Nursing, especially in Private Practice, was delivered to Nurses at St. George's Hospital on Thursday, January 6, by Sir William H. Bennett, K.C.V.O., F.R.C.S.

Sir William said:—Miss Smedley, Sisters and Nurses,—When requested to address you this evening my first inclination was to decline, because I did not quite see how I could usefully say anything of interest, seeing that of course you have already received all possible instruction as to the various details of nursing from others who are more in touch with the elementary parts of the subject than I am myself. On second thoughts, however, it struck me that it might be of some advantage if I were to say something concerning a matter of which I have an extremely intimate knowledge, namely, that of nursing in private practice. It is often said that "lookers-on" see most of the game and you must understand that the remarks which follow are based upon what I have actually seen of nursing and nurses in the course of a large experience. It is unnecessary I suppose to point out that the majority of you who are present to-night, although occupied in hospital work at the present time, must sooner or later seek some other fields for your efforts, seeing that the opportunities offered by hospital work, either here or in other institutions, must of necessity be small compared with the number of you who are engaged in your education at the present time. That being the case, the question arises as to what is to happen to you at the end of your hospital career, that is to say, after the time at which you obtain your ordinary certificate of training in St. George's Hospital. Three courses suggest themselves obviously at once: the continuation in hospital work, either here or in other institutions, supposing that you find opportunities for doing so; and if such appointments come within your grasp, it is my candid advice to you to take advantage of them if you can. Some of you, who are of a sufficiently speculative turn of mind, will, I suppose, marry; a course which naturally will remove you from the ordinary routine of nursing work. But above and beyond those of you who can possibly find employment, either in this hospital or in other similar institutions permanently and those who will marry, there must be a large number of you who will be thrown upon your own resources in order to obtain a livelihood in the profession which you have adopted. In other words, a large number of you, if you are to continue your work at all, will have to adopt what is commonly called the practice of private nursing. That being the case, although the remarks which follow will have some general application, they must be held to more especially apply to those of you who will finally find yourselves occupied in the nursing of private patients.

### The Working Life of a Nurse.

To begin with, it should be remembered that the working life of a nurse is short, and it is therefore of vast importance that you should weigh carefully what course you are to adopt in the future. During a very large experience of surgical work I do not recollect to have met in the ordinary course of my practice more nurses than I could count on the fingers of my two hands who had passed much beyond the age of 40, and it would be interesting to know what happens to the majority of nurses after that period of life. It is abundantly evident that my experience in this respect is not peculiar, for there has within the past two years or so been established an institution upon the co-operative principle exclusively for nurses who are more than 40 years of age. Here let me remind you that those who are engaged more particularly in surgical nursing have the shortest life of all in the matter of their work, for the obvious reason that in the nursing of surgical cases there is required an amount of energy, of physical strength, and of general resource which is in excess of that which is as a rule necessary for other nursing work. In maternity nursing, the age at which a woman may follow her occupation is practically unlimited. So that you have, to start with, the two extremes; the surgical nurse on the one hand, who represents the shortest working life of the series; the obstetric or monthly nurse on the other hand, who represents the longest of the series; and, intermediate between these two, is the nurse who is more especially concerned with medical cases, the calls upon her resources being intermediate between those which are made upon her in the two other classes which I have mentioned. The fact that there is this difference in the various departments of nursing makes it obviously desirable that the nurse should not specialise to any great extent, but that she should become what would be called in my own profession a good all-round general practitioner, in order that when the calls upon her strength and resources—in connection, for example, with surgical work—are more than she is quite justified in undertaking, she may be in a position to fall back upon one of the other classes to which I have referred, in order to prolong the period of her working life. I therefore strongly advise all those of you who can do so to take out a course of education at some well-recognised lying-in institution and to obtain such certificates or diplomas as are accessible to you in connection with obstetric work. For the same reason it is extremely desirable that you should have a sufficient acquaintance with massage to undertake some of the more simple applications of that treatment so often required during the convalescent stages of cases which you have been previously nursing.

### The Education of the Modern Nurse.

It is hardly needful to say that one of the first essentials for a nurse is a sound mind in a healthy body; and I assume that those who have passed through the training at a hospital like this have, by the time they have arrived at the conclusion of that test, been able to come to the decision as to whether they are sufficiently sound in body and stable enough in mind to continue with the practice of their calling. I am, therefore, now speaking more especially to those of you who, having passed through the ordinary training period, find yourself at the end of that time strong in body and healthy in mind, ready to face the trials and anxieties which naturally attach to the calling which you have adopted. The education of the modern nurse has been developed to a remarkable degree, and the methods of education, although they differ to a certain extent at the various institutions, all tend in the same direction, that is to say, towards the improvement and general elevation of the nurse's knowledge of anatomy and physiology apart from the special details connected with nursing itself. Now this evolution—for it is an evolution and nothing else—has obvious and great advantages, and at the same time it has its disadvantages, because with the increased knowledge of matters collateral, although they may be more or less nearly connected with the subject of practical nursing work, there is naturally a temptation to regard the real essentials of nursing as being perhaps rather too commonplace to receive quite the serious degree of attention which they should do. Moreover the increase of responsibility which has come to devolve upon the nurse at the present time tends, human nature being what it is, rather to an exaggerated sense of the importance of the individual in relation to little details. The result of this, what I am almost inclined to think is in one direction excessive education, is sometimes to produce a class of nurse which, in my opinion, is not of the best type, whose existence therefore is not to the advantage of the nursing profession generally. This, I think, is partly, as I have already intimated, the fault of the educational methods sometimes employed. It does not, for example, to me appear to be any great gain to a nurse that she should possess such a knowledge of physiological chemistry, for an example as would acquaint her with the changes which take place in the food as it passes through the stomach, if it leads in any way to detract attention from the value of the nurse's true function, and I have known cases in which nurses, highly educated from this point of view and who seemed to have had a certain knowledge—superficial, of course, but still a certain knowledge—of anatomy and physiology, have proved anything but what I call nurses in the ordinary meaning of the term. I may, perhaps, be allowed here to digress for one moment to impress upon you what a nurse really is and what the objects of her work are.

### The Nurse and Her Work.

A nurse is a person who is deputed under authority to administer to and to care for a sick person. The bulk of her duties is concerned with manual—you may call it, if you like, manipulative—work which tends to the comfort of the patient, and, aided by such moral influences as she is able to exert under ordinary circumstances, has a large share in recovery after operations, and in illness generally. Now, a highly-educated nurse in the transcendental sense is not always an efficient nurse from the point of view of the surgeon, the physician, or the practitioner. There comes to my memory, for example, an instance in which a nurse, after an operation, occupied herself in describing to the patient the anatomy of the part concerned with a certain degree of accuracy. But that knowledge did not prevent the application of a hot-water bottle in such a way that mortification followed its use. I only mention this to show that a highly-educated nurse, in the sense in which the term is sometimes, and I think unfortunately, used, is not necessarily efficient in real nursing work, and, indeed, sometimes may be distinctly defective in a knowledge of its practical details, with which, of course, she ought to be intimately acquainted. It cannot be amiss to emphasise here the fact that the work of the nurse is hard; the labour itself is considerable; the physical strain is sometimes very great; the mental strain is sometimes more; and it is well that those who are yet in the early stages of training should dismiss from their minds altogether the idea that there is anything romantic or idealistic about the profession of nursing. You have adopted a calling which is sometimes called noble; and noble no doubt it is supposing that it is carried on in the right way by those who practise it. But the fact of that name having been applied to the calling does not remove the practical truth that the greater part of the work is menial and commonplace, and that you will find, noble as the calling may be, that it will tax your resources to the utmost if you are to make a living in the ordinary sense of the word whilst you are engaged in it.

### The Refinement of the Modern Nurse.

Following upon the question of education another point arises in connection with the modern nurse. Speaking generally, there is no doubt that the nurse of to-day is in some respects very superior to those of the recent past; she is more refined, not only in consequence of her superior training in the wider educational sense, but also because she is generally drawn from a higher class of the community than was the nurse of 25 or 30 years ago; with refinement in the individual there is sure to follow, to a certain extent, refinement in her methods. This in the main, no doubt, is advantageous to the nurse and to her calling. But this very refinement and this very increase in the degree of education to which I have already referred, tend I fear occasionally somewhat to the disadvantage of patients. The existence of this refinement, there is no doubt, leads at times to a shrinking on the part of nurses from thoroughly dealing with the management of certain cases. There are, of course, as you must know, because all of you here to-night have had a certain amount of experience, some cases of disease affecting certain parts of the body which I need not particularise, as you must know full well what I mean, which are naturally more or less distasteful under any circumstances to nurses generally; and the more refined the nurse and the more highly educated she is, the more this inclination to avoid the actual contact with cases of this kind shows itself with the majority, and it is not surprising that this should be so. Now, it must be distinctly understood that, so far as the nurse is concerned in private work, every case of disease or sickness is a case with which she must be prepared to deal. It is not possible—at all events if it is possible it is not practicable—for a nurse to select her cases, or to decline to undertake any duty however unpleasant which arises in connection with them. The sooner you understand that you must be prepared, if you are to follow your calling with any credit to yourself and to it, not to allow any sense of false delicacy to interfere with your dealing with any case, no matter what it is, which is placed before you, the better it will be for yourselves and the better it will be for your calling.

### Relation of Nurse to Patient.

With regard to disease affecting certain parts, you must understand that in the relation of nurse to patient in sickness there is no such thing as "decency" or "indecency." A very old lady, a true woman of the world, a great friend of mine, I remember quite well rebuking a child on one occasion who, in speaking of the scanty dress of some person, said: "Oh, I think it was quite indecent." "My dear," said the old lady, "when I was your age," the age of the child was about ten, "the words 'decent' and 'indecent' were not in my dictionary." And I would commend to you as nurses with regard to your patients the observation of my old friend to the child under the circumstances which I have mentioned. In connection with this matter it is a pleasure to be able to say that recently I have been concerned outside the hospital with a case in which an illness of the kind under discussion arose, in which the nurses who conducted the case came from a hospital where I am told care is taken that the nursing of cases of this class is conducted to a large extent by male attendants in order to relieve the ordinary nurse from the necessity of dealing with them. It may perhaps at first sight seem under the circumstances surprising that I am able to say that I have never seen any case of the type better nursed or more thoroughly dealt with than this one was—which says a great deal for the institution from which these nurses came, and it says a great deal more for the nurses themselves. What I want you especially to bear in mind is that if you are to engage yourselves in private nursing, you will have to undertake any duties which are set down to you by the surgeon, physician, or practitioner by whom you are employed, that you will not be in a position to select your cases or your duties if you are to follow your calling with real advantage to yourselves.

*(To be concluded.)*

### The State Registration of Nurses.

A SPECIAL general meeting of members of the Royal British Nurses' Association was held at the rooms of the Medical Society of London on Friday last, for the purpose of discussing the recommendations of the Executive Committee as to the presentation to the House of Commons of a Bill "for the State Registration of Nurses." The Chairman was Mr. Pickering Pick. Among those present were Mr. John Langton, Hon. Treasurer; Dr. T. Comyns-Berkeley, Hon. Medical Secretary; Miss Hobbs, Dr. and Mrs. Bedford Fenwick, a large number of nurses, and others. A synopsis of the proposed Bill drawn up by the Executive Committee was placed in the hands of all present, and the various clauses were put to the meeting seriatim, and after some discussion were finally adopted with but few amendments. The principal were as follows:—The addition to the Central Board of a member of the Asylum Workers' Association to take the place of one of the two Provincial Medical Practitioners to be appointed by the General Medical Council. This was proposed by Mr. Langton and carried unanimously. On the suggestion of Dr. Heron, the addition was also made to the Central Board of a member of the British Medical Association. This increased the number of registered medical practitioners to ten, and it was felt that the number of matrons should be proportionately added to; it was therefore decided to add a member of the Matrons' Council. Beyond a slight alteration in the arrangements for the retirements of members necessitated by this increase in numbers, these were the only important amendments. A motion for the doubling of the number of fully-trained nurses on the Board—six—which received the support of Mrs. Bedford Fenwick, was put to the meeting and lost by a large majority. Mrs. Bedford Fenwick also criticised unfavourably the appropriation of nurses' fees to the general expenses of the Board.

The proposals contained in the synopsis as finally agreed to are as follows:—

That a central board shall be created and shall consist of ten registered medical practitioners appointed for three years and eligible for re-election; one to be appointed by the Royal College of Physicians of London, one by the Royal College of Surgeons of England, one by the College of Physicians and Surgeons of Edinburgh, one by the College of Physicians and Surgeons of Ireland, one by the Society of Apothecaries, one by the Royal British Nurses' Association, one by the Incorporated Midwives' Institute, one provincial registered medical practitioner to be appointed by the General Medical Council, one member of the British Medical Association, and one member of the Asylum Workers' Association. Three lay members appointed by the Lord President of the Council to represent England, Scotland, and Ireland. Three nurse representatives, one representing the army and navy to be appointed by the Directors-General of the two services, one to be appointed by the Royal British Nurses' Association, and one by the Queen Victoria Jubilee Institute for Nurses. Ten fully-trained nurses who are matrons or lady superintendents of hospitals or infirmaries with training schools attached, to be elected by the matrons of the hospitals and infirmaries in the respective areas represented—two to represent the Metropolitan hospitals, one Metropolitan Poor-law infirmaries, two Provincial hospitals, one Provincial Poor-law infirmaries, one each to represent Scotch, Irish and Welsh hospitals—all the above having training schools attached, and one member of the Matrons' Council. Six fully-trained nurses to be elected by nurses on the State Register, resident in the respective areas represented; two to represent the Metropolitan area of London, two to represent the Provinces, one to represent Scotland and one to represent Ireland.

That at the third year from the date of the Act coming into force, a certain proportion of each class of the elected members of the Board shall retire annually and be eligible for re-election. The nurses will be required to pay fees for examination and registration, which will be devoted to examination, certificate expenses, and to the general expenses of the Board. The Board is to publish an annual financial statement certified by an accountant.

That any nurse who, within one year from the date of the Act coming into force, claims to be entered on the Register shall hold a certificate of at least two years' training in an institution recognised by the Central Board, or produce evidence satisfactory to the Board that at the passing of the Act she has been for at least five years in *bonâ fide* practice as a trained nurse, and that she bears a good character. That at the expiration of the time of grace every nurse shall have three years' training, and no nurse shall be registered under the age of twenty-four years. That all nurses shall produce certificates of having received training in medical (including enteric fever) and surgical nursing, and in invalid cookery and sanitation. It is further advisable that all nurses should have had practical training in the other infectious fevers for at least three months.

That certain powers shall be conferred on the Central Board, including power of approving certificates and regulating the conditions of admission to the roll of nurses, regulating the course of training, the conduct of examinations and the remuneration of examiners, regulating the admission to the roll of women already in practice as nurses at the passing of the Act; regulating, supervising, and restricting within due limits the duty of nurses, deciding the conditions under which nurses may be suspended, appointing examiners, deciding upon when and where examinations shall be held, publishing annually a roll of nurses who have been duly certified under the Act, deciding upon the removal from the roll of the name of any nurse for disobeying the rules and regulations laid down by the Board or for other misconduct and also for deciding upon the restoration to the roll of the name of any nurse so removed, issuing and cancelling certificates of registration and inspecting and registering all private nursing homes.

That nurses shall be entitled to wear a special badge and that any unauthorised person wearing the same shall be liable to prosecution. Power is sought for the Board for registering any nurse who has been trained and examined in any part of the British dominions beyond the seas, provided the certificate is satisfactory and that she passes an examination approved by the Board; also for the appointment of a secretary and other officers—the secretary to have the custody of the roll. A copy of the roll of nurses is to be evidence in all courts that the nurses therein specified are certified under the Act.

Any nurse who procures or attempts to procure a certificate by means of a false or fraudulent declaration, certificate, or representation, and any person wilfully making or causing to be made any falsification in any matter relating to the roll of nurses, shall be guilty of a misdemeanour, and shall be liable to imprisonment with or without hard labour for a period not exceeding twelve months.

The synopsis having been agreed to, Dr. Bedford Fenwick congratulated the Association on having at last undertaken the duties for the purpose of performing which it had originally been founded. Dr. Fenwick proceeded to state that when in 1895 the British Medical Association passed a resolution in favour of the State Registration of Nurses, it was opposed by a member of the Royal British Nurses'

Association, and that the Executive Committee of the Association further passed a resolution to the effect that such registration would be inexpedient in principle, injurious to the nurses, and of doubtful public benefit. Dr. Fenwick then asked if this resolution had been rescinded.

Dr. Comyns-Berkeley replied that the Executive Committee had passed a resolution in favour of the State registration of nurses, but that the previous unfavourable resolution had not, to his knowledge, been rescinded.

On the motion of Dr. Comyns-Berkeley it was then resolved that authority be given to the Executive Committee to draft a Bill for the State Registration of Nurses based upon the synopsis presented to the meeting.

### The Nurses' Bookshelf.

HINTS ON HOW TO START A DISTRICT NURSING ASSOCIATION. By a COUNTY SUPERINTENDENT. (The Scientific Press, Limited. 20 pages. 1s. net.)

THIS little book gives in a concise shape hints on the forming of a district nursing association, and may be safely recommended as a guide to those who embark on such an undertaking. It is one to be approached by no means lightly or unadvisedly, but it is a good work, and meets a need that no other effort can. There is no mention in the book of the advisability of insisting upon the medical man's sanction before a call is made on the nurse. It seems desirable that some such rule should be made and enforced except in urgent cases, because the poor are very exacting and somewhat selfish; rendered so, poor things, very often by the hard struggle to live. There might possibly be some friction caused on this point in consequence of subscribers of 2s. and upwards annually feeling themselves aggrieved if they were not permitted at all times to call upon the nurse as their undoubted right. In the matter of sitting up at night with the sick, it would be better to insist upon a written note giving sanction to this arrangement from the doctor in attendance. As night work unfits the district nurse for her daily work, there should be as little of it as possible. The remarks in the section "Lodgings for the Nurse" are most sensible. She never should live alone and depend upon herself for food and attendance.

THE EXAMINATION OF THE URINE. By J. K. WATSON, M.D. (London: The Scientific Press, Limited. 1903. Pp. 30. Price 1s.)

THIS practical little handbook consists of three parts which deal respectively with the anatomy of the urinary organs, the physical characters of the urine in health and in disease, and the collection and examination of the urine. Such an arrangement has many advantages. It renders possible an intelligent appreciation of what the examination of the urine means and why it is necessary at all. The nurse who is merely taught a certain number of tests for albumin, sugar, and so forth, without having any proper understanding of what she is doing or why the tests are required, is pretty sure, sooner or later, to become careless in her methods, and as every nurse must be prepared in the course of her duties to make an elementary examination of the urine, a little book like this is sure to prove valuable. The instruction given is expressed simply and clearly, and the author seems to have found the happy mean between saying too little and too much. The last page is occupied with a table in which are set forth the more important characters of the urine in some of the commoner diseases. An index is also provided to facilitate reference. We have much pleasure in recommending this book for the use of nurses.

### Everybody's Opinion.

[Correspondence on all subjects is invited, but we cannot in any way be responsible for the opinions expressed by our correspondents. No communication can be entertained if the name and address of the correspondent are not given as a guarantee of good faith, but not necessarily for publication. All correspondents should write on one side of the paper only.]

### PIANOS FOR NURSES.

"A LOVER OF MUSIC" writes: In reference to your advocacy of pianos for nurses, may I suggest that pianos in nurses' homes are not an unmixed blessing. My experience is that nine nurses out of ten who perform on that instrument pound and strum so unceasingly that to those who have a sensitive ear and a cultured musical taste the hours which should be for rest and refreshment become a farce.

### PRIVATE NURSING IN MADRAS.

MRS. BROADFOOT writes from Chingleput, Madras, India, under date of December 9th, 1903: In a paragraph headed "Private Nursing in Madras" you seem to suggest that the Nursing Home in Mackay's Gardens has been lately founded by Miss Dent. I founded the institution on February 13th, 1899, and several hundreds of patients had been nursed by me and my sisters and nurses before I handed it over to Miss Dent. It was then in full working order, and I sold the goodwill and furniture to Miss Dent—on my marriage—in April of this year.

### POSTAGE TO THE RIVIERA.

MISS A. BRYANT, matron of the English Nurses' Institute, Sunny Bank, Borgo Pescio, San Remo, writes: May I call attention to the importance of putting proper postage to letters sent me and of enclosing stamps for reply. Although English stamps cannot actually be used for the purpose, they are always useful in other ways. The postage is 2½d. On an average I receive letters asking for a reply every day during the winter months. Most of them come with 1d. stamps, for which I have to pay 5d., and hardly any enclose stamps. This forms a serious item of expense during the season, besides being a disappointment to nurses who are hoping for an answer. I am sure I am only one of many matrons who are out of pocket from this cause.

### STATIONARY SISTERS.

"MATRON" writes: In this infirmary of 700 beds the sisters change their wards about once in twelve months, and have done for the past few years, the system working well. They come on the understanding that they are to serve the institution as required, and are told at the beginning that they will get experience in male, female, and children's wards, also in night duty, the last only if actually necessary. The points in favour, besides the extended experience of the sisters, are that all wards are kept equally well managed down to the smallest detail, that favouritism is unknown, that egotism—"My ward and my patients"—is less known, and that the sisters are not such "personages" as they sometimes quite unwittingly become under the statutory system. I say this last very guardedly, but quite fearlessly, as we all know well that there are many hard working and zealous sisters toiling quietly for years in the same ward and giving that part of the hospital a high tone to the complete satisfaction of the authorities. During my experience I do not think that matrons are averse to change, but doctors sometimes are—for the sister acts as an "aid to memory" to them, and carries out all their "ways"—thus enabling them to accomplish more work in a short time.

### POOR-LAW SUPERINTENDENT NURSES.

"AN IRISH TRAINED NURSE" writes: May I make a few remarks in regard to "An Irish Superintendent Nurse's" letter? She begins by stating that in Ireland Poor-law nurses are now independent of the master and matron, and are subject only to the medical officer. She is mistaken in this, however, as the master still has a control in regard to disciplinary matters—a grievance often to nurses, who demur to the views and ways of an often ill-mannered and, in a sense, illiterate man, incapable of appreciating the fitness of things. Yet he is the resident head, and the doctor is not. Officers styled "superintendent nurses" are few and far between in Irish workhouses, and such a title is scarcely known outside a city, and often not even there. May I say that money is not "frequently given in lieu of rations," and that, in my experience, far more than "one case in ten requires real nursing." If they did not, they would not be located in the infirmary proper by the medical officer, but be sent to another portion of the house. "Interesting" cases are unfortunately just as frequent in our union hospitals as in any general infirmary, and should exact, even though they are only God's poor, all the thought, care, and attention of even a "Superintendent Nurse."

### TRAINING UNDER THE ROYAL ARMY MEDICAL CORPS.

"RUTTY" writes: The letter of "Trained Male Nurse" is truly an "attempt" only to prove the superiority of the Royal Army Medical Corps training to that of the National Hospital, even though he allows that the former is not complete. No such haphazard foundation as a "man's luck," upon which to build a sound nursing education, exists at the National Hospital. There is no depending upon superior officers' whims as to granting opportunities for improvement, no drills at the option of that officer to interfere with the nurse's training, no uncertainty as to the class of hospital he is sent to, and indeed no trusting to the chapter of accidents at all. On the other hand, at the National Hospital all facilities and encouragement are not only freely given, but insisted upon. Again, instead of chance determining which cases he may be given charge of, the nurse at the National is bound to go through a methodical training, embracing not only paralysis, epilepsy, and kindred diseases, but also typhoid, pneumonia, pleurisy, and the whole run of general diseases. This disproves "Trained Male Nurse's" comparison of a National training to that of a purely maternity hospital. Moreover, the National Hospital enjoys the services of a distinguished staff of surgeons and physicians. The attendance of the nurse is compulsory at all lectures and operations in the theatre of the hospital, and all the latest discoveries in medical treatment are at the nurse's educational disposal, together with a knowledge of massage, the uses of medical electricity, etc. Moreover, it is only after the National nurse has proved himself thoroughly efficient that he is granted a proper certificate of competency.

### ASSISTANT NURSES IN WORKHOUSE INFIRMARIES.

MISS ANNIE LOUISA LEE, Hon. Secretary of the Meath Workhouse Nursing Association writes: As the subject of the nursing of the sick poor in workhouses has again been reopened in THE HOSPITAL, I think your readers may be interested to know that during the last seven or eight years, upwards of 170 Poor-law appointments have been accepted by nurses of this Association. The large majority of these appointments have been those of assistant nurses in provincial workhouses. But amongst them are several superintendent and staff nurses. The large demand for assistant nurses continues, and we have been unable fully to meet it. On the other hand, we have not always found it easy to place our fully-trained nurses in Poor-law appointments. The demand, as far as our experience goes, is for assistant nurses—that is, nurses of proved character who have had one or two years' institutional experience and training. Possibly your readers might be able to give me information as to whether the need for fully-trained nurses is now met by the nurses from these infirmaries which are recognised training schools? If so, we should be glad to concentrate our efforts on training assistant nurses to work under them in provincial or other workhouses. I hope that we may receive from your readers some information as to the nurses provided by these infirmary training schools.

### POOR-LAW VACANCIES AND GENERAL HOSPITAL-TRAINED NURSES.

"AN INFIRMARY-TRAINED NURSE" writes: Will you allow me just to say a few words with regard to hospital and infirmary nurses. I think that it is rather a mistaken idea to say that hospital nurses obtain all the best of the appointments, or that infirmary nurses are never given hospital posts. Many good appointments are given which are never published, owing to the fact that many nurses do not trouble to send in a notice to the nursing papers. I can speak from experience, as I received my training in one of the largest London infirmaries, and have since held higher appointments both in another London infirmary, and also in a hospital, and I have never experienced the slightest difficulty in getting on. Several nurses who trained at the same time as myself afterwards obtained very good posts in general hospitals, and several nurses who were trained in the last infirmary I was in are holding good posts in hospitals at the present time. I myself am deeply grateful to hospital-trained sisters for many things I have learnt, and I am sure many hospital-trained nurses will not be too proud to admit that there are some things they have been able to learn even from an infirmary nurse. We must remember that however proud we are of our infirmary training schools they have not always been what they are at the present day, and that it is due in a great measure to the untiring energy and devotion of hospital men and women that they have been brought up to their present standard of excellence. Seeing that we owe them so much in the past we should not wish to debar them from applying for our posts and taking equal chances with us, especially as the hospital authorities are willing to allow us to compete with their nurses. I think to a very great extent it rests with ourselves whether we do well or not, provided of course that we have our three years' certificate of training, without which it seems almost impossible to do much at the present time.

A CRUMPSALL NURSE writes: May I be permitted to answer "One of the Staff" through your valuable paper, and to prevent her in future from "displaying her ignorance" *re* the training of a workhouse hospital nurse. Your correspondent evidently is not aware that union hospitals containing the required number of beds, resident doctor, lectures, etc., are as much recognised by the Local Government Board as training schools as are voluntary hospitals and infirmaries. "One of the Staff" has never visited a union hospital containing about 1,000 beds, and certainly never worked in one. I am sure she would be "at sea" and "feel her position" for a time, were she so lucky as to be appointed Sister; also she would find her equals and superiors both professionally and socially in that institution. I have no doubt that when "One of the Staff" has gained more experience she will be a little more broad-minded, and learn to regard her fellow-nurses in a more friendly spirit.

"A NURSING SISTER" writes: It has greatly grieved me, and doubtless many others, who love our profession, and would fain see its high calling held in good repute, to read the controversial statements in the last few issues regarding "Hospital" *versus* "Poor-law Infirmary Training." Surely, if we would all remember that "noblesse oblige," and that "unity is indeed strength," we should look with scorn at the absurdly petty details which seem unaccountably to have fixed so wide a gulf between "hospital-trained" and "Poor-law infirmary trained" members of a mighty force of ministering women, approved of and certificated by qualified members of the medical profession, and by matrons of undoubted experience in our capabilities. Proud we are, one and all, of our training school, and loyal to all who have benefited us in it, by theoretical or practical assistance; but I take it that in the case of the sick (either in institution, private, or district nursing) a three years' certificated and conscientious nurse is nowadays equally capable, whether she hails from "Poor-law" or "Hospital" work. "Hospital" was the nursery, in which the profession of trained nurses had its birth, but it has been strengthened in its adult stage, by the practical teaching in many details of Poor-law work. Then may I touch upon the friction, hinted at by an "Ex-Matron" in her article on the difficulty of filling a "night sister's" post. Again the oneness of our object, and in addition the desirability of illustrating this fact to our probationers comes before us, as "Ex-Matron" places to the fore, as an obstacle, and apparently a very "thorn in the flesh" to the night sister—the day sisters and their "clannishness." Her experience with the latter must have been unfortunate, and though I

grant not unique, I maintain that by the exercise of tact and by keeping our high standard well in view, day and night sisters may work in absolute harmony, and so make the work a continuous chain instead of an undoing of one another's labours, to the detriment of the patients' comforts and morals, the upsetting of the ward discipline, the addition of extra worry to the mind of an already busy matron, and last, but not least, to the wreck of our hope, to show our probationers, by example, as well as by precept, that "charity" is the keynote of a nurse's calling. "Ex-Matron" appears to have had an unusual experience with her night sister, as she implies, that "never seeing the house physician," no "night round," on which the night sister would surely accompany him, was done by her resident medical officers. That, too, seems strange, when we all admit that it is at eventide and in the silent watches of the early dawn that a crisis often comes or that life is at its lowest ebb. I think that in most institutions the night sister has her opportunity before the doctors retire to rest of bringing to the notice of one or another of them any bad cases under her care and of a little social intercourse with them before her night watch begins. Doubtless the strain is great, especially to one who cannot turn day into night; unquestionably, too, the responsibility of acting for herself or of summoning medical assistance weighs heavily at times upon her, but I submit to any of my fellow-sisters who have taken both duties that each one, either day or night, has its trials, and each its unasked-for reward, and that each may by little acts of thoughtful kindness lighten, instead of add to, one another's burdens.

### Novelties for Nurses.

BY OUR SHOPPING CORRESPONDENT.

### THE DRIPLESS CUP.

EVERYONE knows the disagreeables of a dripping cup. It cannot be avoided when there is liquid in the saucer and the ordinary cup is used. But the disagreeable is banished if a dripless substitute, as supplied by the firm of J. N. Masters, of Rye, is used. The base of the cup is a circle of little arches so close together as to allow the cup to stand firm, and yet avoid a rim of moisture to develop into the unpleasant drop, so ruinous to clothing and temper. No extra expense is incurred by adopting this improvement, and Mr. Masters has china of various quality and design, from which a selection may be made.

### AN EMERGENCY GOWN.

I HAVE before me a gown which combines the appearance of a dress with the convenience and warmth of a dressing-gown. Such a combination will appeal to those who are liable to be aroused suddenly, with the probability that no time will be available to make a complete toilet, possibly for some hours This gown has been contrived and is supplied by The Nurses' Co-operative and Outfitting Association, Wellington Road South, Stockport, who supply it in several qualities. The better gown is made of a soft grey woollen material which is washable and claims to be unshrinkable. The garment is adjustable in a minute, and it is so fastened that the wearer has the appearance of being completely costumed. The warm lining renders it substantial and comfortable. The Association is recommended for its dress-making, and is very successful in fashioning garments from given measurements.

### To Nurses.

**WE invite contributions from any of our readers, and shall be glad to pay for "Notes on News from the Nursing World," or for articles describing nursing experiences, or dealing with any nursing question from an original point of view. The minimum payment for contributions is 5s., but we welcome interesting contributions of a column, or a page, in length. It may be added that notices of appointments, entertainments, presentations, and deaths are not paid for, but that we are always glad to receive them. All rejected manuscripts are returned in due course, and all payments for manuscripts used are made as early as possible after the beginning of each quarter.**

### Appointments.

[No charge is made for announcements under this head, and we are always glad to receive, and publish, appointments. The information to insure accuracy should be sent from the nurses themselves, and we cannot undertake to correct official announcements which may happen to be inaccurate. It is essential that in all cases the school of training should be given.]

AXMINSTER COTTAGE HOSPITAL.—Miss Rachel C. M. Reid has been appointed matron. She was trained at St. Thomas's Hospital, London, and has since been sister at the Hospital for Women, Shaw Street, Liverpool, and sister in Deaconess Hospital, Edinburgh.

BLACKHEATH AND CHARLTON COTTAGE HOSPITAL.—Miss Mary Stanford has been appointed matron. She was trained at Brompton Consumption and St. Thomas's Hospitals. She has since been staff nurse at University College Hospital, London, ward sister at Bristol Royal Infirmary, and in charge of a West End Nursing Home.

BRISTOL ROYAL INFIRMARY.—Miss M. Bewsher has been appointed sister. She was trained at the Cumberland Infirmary, Carlisle, where she has since been theatre sister.

LONDON OPEN-AIR SANATORIUM, WOKINGHAM, BERKSHIRE.—Miss Amy Bullock has been appointed matron. She was trained for one year at All Hallows' Hospital, Ditchingham, Norfolk, three years at the Grosvenor Hospital for Women and Children, Vincent Square, Westminster, and three years at the London Hospital, Whitechapel. She has since been nurse-matron, *pro tem.*, at the Infirmary, St. Nicholas Industrial School, Manor Park, E., assistant-matron at St. Mary's Infirmary, Islington, home sister and housekeeper at the Royal Hants County Hospital, Winchester, and lady superintendent and secretary for the Ockley Benefit Nursing Association, Nurses' Hostel, Horsham.

NEWCASTLE-UPON-TYNE UNION HOSPITAL.—Miss J. Pauline Kelsey has been appointed charge nurse. She was trained at the Bethnal Green Infirmary, London.

ROYAL VICTORIA HOSPITAL, BOURNEMOUTH.—Miss Florence Alex Harris and Miss Katherine Thorburn have been appointed charge nurses. Miss Harris was trained at the Newport and Monmouthshire Hospital, where she was afterwards sister. She has since been senior staff nurse at the Cottage Hospital, Bromley. Miss Thorburn was trained at the Royal Derbyshire Infirmary.

### Presentations.

ROCKFERRY NURSING INSTITUTION.—On Christmas Day, Miss Prince, matron of the Rockferry and Liscard Nursing Institution, Cheshire, was presented, by her staff, with a handsome inlaid solid oak frame grandfather clock.

CUMBERLAND INFIRMARY, CARLISLE.—Miss M. Bewsher, who is leaving Cumberland Infirmary to take up the duties of sister at Bristol Royal Infirmary, has been presented by the matron, sisters and nurses with an afternoon tea service and silver tea spoons.

### Wants and Workers.

WILL anyone forward THE HOSPITAL when done with, for payment of postage, to District Nurses, Wallsend-on-Tyne.

A MIDWIFERY NURSE would be very grateful for linen or left-off underclothing, for poor patients. Nurse L. O. S., "Rosville," Great Shelford, Cambridgeshire.

### Echoes from the Outside World.

### Movements of Royalty.

THE King and Queen brought their visit to Chatsworth to a close on Monday morning, the King travelling direct to St. Pancras, and the Queen, with Princess Victoria, returning to Sandringham. The weather, which during most of the Royal visit was unfavourable, greatly improved on Saturday, and a party, including their Majesties, were conveyed in three motor cars to Hardwick Hall. Passing through Chesterfield they were received with enthusiasm, and the town had been decorated for the occasion. Luncheon was served at Hardwick, and an hour or so was devoted to the inspection of the interesting old mansion, the party returning to Chatsworth shortly before five. Rain and snow fell so heavily on Sunday for a couple of hours, that the King and Queen did not, as arranged, attend Edensor Church in the morning, but later in the day they were able to go out, and in the evening they were present at Divine Service in the private chapel at Chatsworth. On Tuesday the King rejoined the Queen at Sandringham.

### The Garter King of Arms.

THE death of Sir Albert Woods, Garter Principal King of Arms, in his eighty-eighth year, was announced on Friday, and on Monday he was buried. The news was immediately telegraphed to the King and Queen and other members of the Royal Family, also to the Earl Marshal. Sir William succeeded his father in 1838 as Garter King of Arms, being advanced to the office of Garter Principal King in 1869. Since 1865 he had been attached to all Garter missions to foreign monarchs. In 1865 he attended the investment of King Charles IX. of Denmark with the Order, in 1866 that of King Leopold II. of Belgium, in 1867 that of Emperor Francis Joseph of Austria, in 1878 that of King Humbert of Italy, in 1881 King Alphonso XII. of Spain, and in 1892 that of King Albert of Saxony. Sir Albert was present on duty at the coronation of Queen Victoria, and was consulted as regards the preparation for the coronation of King Edward VII. in 1902.

### Battle in Somaliland.

ON Tuesday the Secretary of State for War issued a report from General Egerton, dated from Jidballi, January 11th, that the British advanced on the 10th against a force of some 5,000 Dervishes who were holding Jidballi. The Dervishes advanced to charge, but could not face the frontal fire of the infantry and the flank attack of the mounted troops. They broke and fled, pursued for ten miles by the mounted troops. The enemy lost about 1,000 and many prisoners were taken. On the British side two officers—Lieut. Charles Henry Bowden-Smith, and Lieut. J. Raboteau Welland, M.B.—were killed and nine wounded, another, Captain Lister, Lord Ribblesdale's heir, being missing. Nine natives and irregulars were killed and twenty-two of the rank and file wounded.

### Boiler Explosion on a Warship.

THE Secretary of the Admiralty received a telegram from Sydney last week stating that there had been an explosion on H.M.S. *Wallaroo*, with 43 killed and wounded. Later telegrams showed that the accident was less serious than at first appeared, as the signal had been misread. The number should have been given as "four killed and three injured," but through an error the two figures had been put together. The cause of the disaster is unknown. No noise was heard by those on deck, but suddenly steam was observed issuing from the stoke-hole ventilator. Several men tried to ascertain the cause of the escape of steam, but owing to the denseness of the vapour they were unable to descend for some time. Meanwhile, one of the poor fellows, dreadfully burned about the face and stone blind, ran up on deck and almost immediately dropped dead. Two others who were also very badly burned, died about an hour after being rescued. The fourth man was ultimately found in the stoke-hole floating in two feet of scalding water, the boiler having emptied itself into the stoke-hole. Two of the injured men are progressing favourably, but the third was more seriously hurt, and died a few days later.

### Entertaining the Deaf and Dumb.

A NEW YEAR'S tea-party was given at the Holborn Town Hall, last Friday, to 730 deaf mutes. It was a particularly cheerful gathering, all seeming very happy and bright, and evidently enjoying to the utmost the entertainment provided for them. In a few cases a small charge had been made, but the poorer guests had received free invitations. The Association which promotes this function annually exists mainly to provide spiritual welfare for the two thousand odd deaf and dumb in the metropolis. After tea a number of speeches were given, the secretary of the Association and his assistants interpreting when needful by means of the finger language with marvellous rapidity. Amongst other "speakers" was Sir Arthur Fairbairn—himself a deaf-mute—Dr. Elliott, the head of the institution at Margate, and Miss Elena Eklund of Finland, a deaf and dumb missionary.

### A Famous American Singer.

THE death of Madame Antoinette Sterling took place on Sunday morning at her residence in Hampstead. Although a native of America, having been born in 1850 at Sterlingville, New York State, Madame Sterling had resided for many years in England. Her first appearance here was in the provinces, but she went to America again in 1871. There she met with great success as a concert singer. In November, 1873, she sang for the first time in London at one of the promenade concerts at Covent Garden Theatre, and soon became exceedingly popular. Endowed with a rich contralto voice, and careful to distinctly enunciate her words, she achieved her greatest success in Old English songs such as "Three Ravens," "The Three Fishers," and "The Lost Chord." In 1893 she went for a tour in Australia and was well received. In 1875 she married Mr. John MacKinlay, and one of her two children is a well-known baritone vocalist. Madame Sterling's remains were cremated at Hampstead on Wednesday.

### International Art at the New Gallery.

FOR the fourth time the International Society of Sculptors, Painters, and Gravers is holding an exhibition in London, but this year its habitation is the New Gallery, Regent Street. The show is specially interesting because it gives an opportunity of comparing methods in vogue on the Continent with English modes. The president this season, in place of the late Mr. Whistler, is M. Auguste Rodin, who was on Tuesday received in audience by the King. He has six specimens of his work on view, including a large plaster cast and a smaller bronze one of "Le Penseur," a head of "Bellona," and "Le Defense"—where a winged figure is striving to stimulate to fresh effort a wounded man. Mr. Lavery's "Lady in Pink" is perhaps the most noteworthy picture in the exhibition, the dress of the subject being marvellously represented. There are three not specially happy examples of Mr. Whistler's style, before one of which hangs a laurel wreath of silver; a refined and beautiful rendering of "Amsterdam,' by Matthieu Maris; "The Dead Child," a powerful plaster group by M. Bartholome, full of tragic despair; "Denil Marin," and "Messe du Matin," by M. Charles Collet—both dealing with the sorrows of Brittany fisher-folk; "The Toilet," a picture by Mr. Charles Shannon, and "Miss Janie Martin" by Sir James Guthrie. None of these should be overlooked.

### The Awakening of a Soul.

"THE STORY OF MY LIFE."*

MARK TWAIN is reported to have said that "the two most interesting characters of the nineteenth century are Napoleon and Helen Keller;" and after reading this remarkable book we are less surprised at the statement.

Deaf, blind, dumb: yet the writer of her autobiography! It is well-nigh impossible for anyone possessing all their faculties to realize that the first half of this volume was written by one deprived of three senses; yet as the letters (1887-1902) which follow are read, and they show the stages by which the child's mind developed, all doubt as to the authenticity of this life story are dispelled.

And a wonderful story it is of triumph over the deprivation and obstacle. Helen Adams Keller was born in 1880, and seems to have been as other infants until an illness at the age of nineteen months left her without sight or hearing. Her parents, well-to-do people living at Tuscumbia, Alabama, were of course much distressed about their unfortunate child; but for a long time there appeared no way of helping her.

From the difficulty of making her understand anything at all she was fast developing into an absolute savage when, at the age of seven, her parents engaged the services of Miss Anne Mansfield Sullivan as her governess. That lady's own account of her first interview and subsequent intercourse with her pupil given in a letter written at the time is interesting, and shows the heart-breaking difficulty of the task she undertook.

It was a case of word upon word, line upon line: here a little and there a little, day after day, month by month. At last the poor child's understanding was reached; her curiosity was aroused, and she began to ask questions. After that it became comparatively easy to teach her. Her brain, once set to work in the right direction, developed rapidly. The acuteness of her sense of touch proved astonishing, even more so than in the case of those who are blind but can hear. Miss Keller herself thus describes how she learned the beauties of nature: "I recall many incidents of the summer of 1887 that followed my soul's sudden awakening. I did nothing but explore with my hands and learn the names of every object I touched; and the more I handled things, and learned their names, the more joyous and confident grew my sense of kinship with the rest of the world. . . . As my knowledge of things grew I felt more and more the delight of the world I was in. Long before I learned to do a sum in arithmetic, or describe the shape of the earth, Miss Sullivan had taught me to find beauty in the fragrant woods, in every blade of grass, and in the curves and dimples of my baby sister's hand."

Everything, of course, is conveyed to Miss Keller by the sense of touch, except as will be observed in the above quotation where she speaks of the "fragrant" woods. Her sensitiveness in this respect is also remarkable. She says that when out in her boat on the lake at Wrentham, Massachusetts, "Nothing gives me greater pleasure than to take my friends out rowing when they visit me. Of course, I cannot guide the boat very well. Someone usually sits in the stern and manages the rudder while I row. Sometimes, however, I go rowing without the rudder. It is fun to try to steer by the scent of watergrasses and lilies, and of bushes that grow on the shore." To some extent, perhaps, Miss Keller's vivid imagination assimilates what she has read or been told about to such an extent that she believes she knows involuntarily what she describes. She mentions the traffic of the streets; and unless the paving of American cities conveys vibration more than does that of London, it is impossible for a totally deaf—and blind—person to have any idea of the din made by passing vehicles. Even so, however, it is marvellous how she realizes things.

The means by which Miss Keller was taught consisted of the finger alphabet, whose signs were made into her own hand by that of her teacher. It was thus that Dr. Howe had rescued Laura Bridgman. But Miss Keller has far outdistanced Miss Bridgman. Having mastered English and become able to read books embossed for the blind, she set her heart upon going to college, on an equality with other young women. This she eventually accomplished, printing her answers to the examination papers on a typewriter.

Miss Keller's brain must be one of the strongest, for—always remembering her difficulties—she has acquired Greek, Latin, French, German. She can even read language from people by placing her fingers to their lips. To a limited extent she has become able to speak. But this, as the editor remarks, "is rather an accomplishment than a necessity;" and it may be interesting to quote what Miss Sullivan writes as to teaching deaf children generally:—"The acquiring of speech by untaught deaf children is always slow and painful. Too much stress, it seems to me, is often laid upon the importance of teaching a deaf child to articulate—a process which may be detrimental to the pupil's intellectual development. In the very nature of things articulation is an unsatisfactory means of education; while the use of the manual alphabet quickens and invigorates mental activity; since through it the deaf child is brought into close contact with the English language, and the highest and most abstract ideas may be conveyed to the mind readily and accurately. Helen's case proved it to be also an invaluable aid in acquiring articulation. She was already familiar with words and the construction of sentences, and had only mechanical difficulties to overcome."

The foregoing shows once again the value of the finger alphabet, the use of which the advocates of the "pure oral," or German system, do all they can to discredit, one result being, as any unprejudiced person who knows anything of the deaf will testify, that those children taught by the manual alphabet grow up far better informed, much easier to have intercourse with, and in every way brighter than those whose teaching has been chiefly concentrated upon the acquirement of speech which only those accustomed to them can understand.

The condition of deaf people would be considerably brighter if more of those possessing all their faculties would take the slight amount of trouble necessary to learn the finger A B C. Yet how rarely, even among doctors, nurses, and clergy can one be found who knows the simple yet magic signs! Some, even parsons, refuse to learn.

Miss Keller well expresses what the state of the deaf is, especially of those situated like herself. Having accomplished so much, she yet feels her loneliness: "Sometimes, it is true, a sense of isolation enfolds me like a cold mist as I sit alone and wait at life's shut gate. Beyond, there is light, and music, and sweet companionship; but I may not enter. Fate, silent, pitiless bars the way. . . . Silence sits immense upon my soul. Then comes Hope with a smile and says 'There is joy in self-forgetfulness.' So I try to make the light in others' eyes my sun, the music in others' ears my symphony, the smile on others' lips my happiness."

Thus with marvellous power of imagination she seeks for patience—a patience learned with so much else from the teacher who, we might say, rescued her from a living death

* By Helen Keller. (Hodder and Stoughton.)

### Notes and Queries.

**FOR REGULATIONS SEE PAGE 135.**

*Electrical Massage.*

(135) I am wanting a book bearing on the subject of electrical massage. It is not a book on "massage" that I want, but on electricity, as it is used with massage. Kindly tell me if there is such a book, and what the price would be?—*M. M. B.*

"Lectures on Massage and Electricity." By Stretch Dowse, M.D. 7s. 10d. post free. It can be obtained through the Scientific Press.

*Books on Infant Management.*

(136) Please give me the name of the best modern standard work for mothers and nurses upon "Infant Management, Feeding, Disorders, Remedies, etc."—*Nurse B.*

"Short Talks with Young Mothers." By C. H. Kerley. 5s. (G. P. Putman and Son). "Health in the Nursery." By Henry Ashby. (Longmans, Green and Co.). "Diseases of Infancy and Childhood." By Henry Koplik. 21s. (Henry Kimpton). "Diseases of Infancy and Childhood." By L. Emmett Hold. 21s. (Henry Kimpton). "The Mother's Help and Guide to the Domestic Management of her Children." By P. Murray Braidwood, M.D. 2s. (Scientific Press). Any of these books can be obtained through the Scientific Press.

*Abroad.*

(137) Will you kindly let me know to whom I should apply for information concerning nursing in France, Paris preferably?—*A. M. W.*

You would obtain reliable information from the Matron, Hertford British Hospital, Rue de Villiers-Levallois-Perret.

*Hospital Training.*

(138) 1. *Miss L.* would be glad to know if the Women's Hospital, Shaw Street, Liverpool, is a recognised training school for nurses. 2. If a lady of 42 could get into an institution to be trained.

1. No. 2. Probably only as a paying probationer.

I desire to become a probationer in a London Hospital. Will you be kind enough to suggest hospitals to which I might apply where no premium is required and where a salary is given from the first.—*L. M. F.*

See "The Nursing Profession: How and Where to Train."

*Health Missioner.*

(139) I wish to become a health missioner to work amongst the poor. I have a certificate for 18 months' general training, and I have been two and a half years in a fever hospital; what further qualifications shall I require?—*Margaret.*

Health missioner is a very vague term, but a knowledge of sanitation and hygiene seems necessary.

*Home.*

(140) Could you tell me of a home where a young woman, aged 24, who has suffered for some years from hysteric epilepsy, could be received? Her mother is in very poor circumstances, and must go out to service as soon as she has found a place for her daughter.—*H. B.*

If the case is hopeless, we are afraid that she will have to be sent to the workhouse, where the guardians will make suitable provision for her welfare.

*Insubordination.*

(141) A nurse, after serving for two and a half years in a recognised training school, left without her certificate on account of insubordination. She has since had three years' training in a good hospital, and would like to know if the former incident would be detrimental to her in applying for a post in the Army Nursing Service Reserve.—*Elba.*

Write and ask the Secretary of the Army Nursing Service Reserve, 68 Victoria Street, S.W.

**Standard Nursing Manuals.**

"The Nursing Profession: How and Where to Train." 2s. net; 2s. 4d. post free.

"Nursing: Its Theory and Practice." (Revised Edition). 3s. 6d. post free.

"Elementary Anatomy and Surgery for Nurses." By William McAdam Eccles, M.D.Lond., M.B. 2s. 6d. post free.

"Elementary Physiology for Nurses." By C. F. Marshall, M.D., B.Sc., F.R.C.S. 2s. post free.

"Ophthalmic Nursing." By Sydney Stephenson, M.B., F.R.C.S., 3s. 6d. net; 3s. 10d. post free.

"Nursing in Diseases of the Throat, Nose, and Ear." By P. Macleod Yearsley, F.R.C.S.Eng., M.R.C.S. 2s. 6d. post free.

"Fevers and Infectious Diseases." 1s. post free.

### For Reading to the Sick.

"HE WHOM THOU LOVEST."

Hushed are all sounds, while like soft mists ascending,
Quiet and calm,
Goes up to Heaven the solemn prayer, portending
Grief's richest balm.

"Lazarus, come forth!" Far down in death's abysses
The glad Soul heard,
And, like a babe new waked by morning kisses,
To life is stirred.

So, without painful change or fearful wonder,
From his calm bed,
Parting the curtains of the grave asunder,
Came forth the dead;

Earnest of that far time when to us waking
This life shall seem,
Amid that higher life upon us breaking,
A strange, faint dream.

*C. L. Ford.*

"Lord, behold, he whom Thou lovest is sick." So spake, by the mouth of a messenger, the sorrowing sisters to their Lord; not dreaming how far more he knew than they did, of Lazarus and his sickness; not guessing that His presence had been with him through all those weary hours of pain; that His love alone had calmed the throbbing heart and soothed the aching brow; that He Himself would bear their loved one, with strong and tender hand, through the valley of the shadow of death.

"Lord, behold, he whom thou lovest is sick."

So perchance may the angels who minister to us on earth address their Lord who sitteth on the throne: guessing but dimly, even amid the light of heaven, how far nearer is the Son of Man to His brethren on earth than ever they can be: not fully knowing even yet how tenderly He watches them from His heavenly home, how gently "He maketh all their bed in their sickness," how they learn to rest in Him when all other rest is as weariness both to soul and body, how He distils upon them the ineffable calm of a will in union with His will, how He bestows on them "the peace which passeth understanding," how at last "He giveth His beloved sleep."—*M. E. Townsend.*

Saviour, perfect my trust,
Strengthen the might of my faith,
Let me feel as I would when I stand
On the rock of the shore of death;

Feel as I would when my feet
Are slipping over the brink,
For it may be I am nearer home—
Nearer now than I think.

*Carey.*

"Lord, what wilt Thou have me to do?" This is the question for all. Say then: "I am ready; let Him do that which is good. I will not be unwise, but understanding what is the will of God." Our Lord blesses such service, and glory is given to God by every breath, by every twinge of pain. Take this lesson as sent you direct from the lips of our Lord, and allow it to rest quietly with all its sweet encouragement in your mind and heart.—*Anon.*

